# Supplementary material for: Economic uncertainty of pandemic and international airlines behaviour
Source: PLoS One. 2022 May 26;17(5):e0266842. doi: 10.1371/journal.pone.0266842 (PMC9135306; doi:10.1371/journal.pone.0266842)
Supplement: S1 Appendix — (DOCX) [file pone.0266842.s001.docx]

**Appendices**

Table A1: Causality in Mean for Full Sample

|  | $\boldsymbol{H}_{\boldsymbol{0}}$**: EMV_ID does not Granger-cause:** | **Quantiles** | | | | | | | | |
| --- | --- | --- | --- | --- | --- | --- | --- | --- | --- | --- |
|  |  | 1 | 2 | 3 | 4 | 5 | 6 | 7 | 8 | 9 |
| 1 | American | 2.415*** | 1.474 | 1.078 | 0.896 | 1.099 | 0.72 | 0.436 | 0.226 | 0.812 |
| 2 | Ana | 3.562*** | 2.060** | 2.465*** | 1.737* | 1.245 | 0.905 | 0.686 | 0.588 | 0.686 |
| 3 | Anaam | 0.656 | 0.741 | 0.542 | 0.318 | 2.114** | 0.298 | 0.407 | 0.378 | 0.088 |
| 4 | Asiana | 2.122** | 1.223 | 0.841 | 1.3 | 1.033 | 0.78 | 0.472 | 0.244 | 0.084 |
| 5 | Cathay | 1.062 | 1.319 | 1.933* | 2.565*** | 2.012** | 1.665* | 1.223 | 0.741 | 0.464 |
| 6 | China | 0.687 | 0.76 | 0.4 | 10.472*** | 5.542*** | 2.428*** | 0.656 | 0.17 | 0.337 |
| 7 | China east | 1.800* | 1.545 | 1.008 | 1.78 | 1.331 | 1.051 | 0.914 | 0.627 | 0.703 |
| 8 | China south | 2.507*** | 1.638 | 1.221 | 0.951 | 0.872 | 0.813 | 0.549 | 0.282 | 0.097 |
| 9 | Delta | 4.291*** | 3.049*** | 2.035** | 1.398 | 0.951 | 0.749 | 0.649 | 0.664 | 0.906 |
| 10 | Deutsche | 1.987** | 0.944 | 1.231 | 0.743 | 1.194 | 1.268 | 0.93 | 0.775 | 0.472 |
| 11 | Eva | 2.177** | 1.345 | 1.598 | 1.022 | 0.867 | 0.54 | 0.343 | 0.323 | 0.111 |
| 12 | France | 2.881*** | 1.619 | 1.048 | 0.73 | 1.099 | 0.723 | 0.436 | 0.223 | 0.075 |
| 13 | Hainan | 0.306 | 0.972 | 0.762 | 1.258 | 7.403*** | 3.812*** | 1.902* | 0.732 | 0.315 |
| 14 | Malaysia | 2.295** | 1.665* | 1.548 | 1.13 | 1.346 | 0.88 | 0.531 | 0.272 | 0.092 |
| 15 | Qantas | 1.841* | 1.449 | 2.506*** | 1.763* | 1.266 | 0.927 | 0.503 | 0.258 | 0.606 |
| 16 | Shandong | 3.890*** | 1.852* | 1.966** | 1.343 | 1.266 | 0.828 | 0.499 | 0.256 | 0.087 |
| 17 | Singapore | 2.095*** | 1.646* | 1.646* | 0.864 | 0.576 | 0.726 | 0.437 | 0.224 | 0.076 |
| 18 | Southwest | 3.383*** | 1.941* | 1.302 | 0.874 | 0.753 | 0.737 | 0.446 | 0.23 | 1.515 |
| 19 | Thai | 3.736*** | 2.209** | 1.559 | 1.404 | 1.135 | 0.859 | 0.45 | 0.233 | 0.081 |
| 20 | United | 3.529*** | 2.416*** | 1.547 | 1.026 | 0.689 | 0.764 | 0.571 | 1.124 | 0.603 |

***,**,* indicates the rejection of the null hypothesis of non-causality at the 1%, 5% and 10%, respectively. The critical values at 1%, 5% and 10% are 2.33, 1.96 and 1.645, respectively.

Table A2: Causality in Mean for COVID-19 sample

|  | $\boldsymbol{H}_{\boldsymbol{0}}$**: EMV_ID does not Granger-cause:** | **Quantiles** | | | | | | | | |
| --- | --- | --- | --- | --- | --- | --- | --- | --- | --- | --- |
|  |  | 1 | 2 | 3 | 4 | 5 | 6 | 7 | 8 | 9 |
| 1 | American | 2.824*** | 2.738*** | 1.854* | 1.344 | 1.032 | 0.861 | 0.48 | 0.249 | 0.749 |
| 2 | Ana | 1.029 | 1.058 | 1.673* | 1.737* | 1.236 | 0.917 | 1.009 | 0.694 | 0.497 |
| 3 | Anaam | 3.296*** | 1.949** | 1.388 | 0.969 | 1.276 | 1.006 | 0.746 | 0.615 | 0.1 |
| 4 | Asiana | 3.105*** | 2.321** | 1.873* | 1.329 | 0.971 | 0.805 | 0.666 | 0.644 | 0.072 |
| 5 | Cathay | 2.565*** | 2.056** | 1.282 | 1.246 | 1.075 | 1.265 | 1.026 | 0.622 | 0.476 |
| 6 | China | 0.637 | 0.557 | 3.924*** | 4.110*** | 2.693*** | 1.829* | 1.149 | 0.596 | 0.395 |
| 7 | China east | 2.763*** | 1.670* | 1.219 | 1.347 | 1.304 | 1.071 | 0.721 | 1.045 | 1.645 |
| 8 | China south | 2.680** | 1.978** | 1.252 | 1.112 | 0.933 | 1.017 | 0.528 | 0.296 | 0.105 |
| 9 | Delta | 3.477*** | 1.594 | 1.524 | 1.151 | 0.944 | 0.671 | 0.406 | 0.21 | 0.87 |
| 10 | Deutsche | 1.909* | 1.082 | 1.044 | 1.827* | 1.313 | 1.364 | 0.79 | 0.316 | 0.107 |
| 11 | Eva | 1.451 | 3.353*** | 2.523** | 1.866* | 1.407 | 1.045 | 0.92 | 0.676 | 0.155 |
| 12 | France | 2.132** | 1.381 | 1.275 | 1.055 | 0.836 | 0.854 | 0.954 | 0.517 | 0.161 |
| 13 | Hainan | 2.102** | 1.745* | 2.154** | 1.696* | 1.271 | 1.03 | 0.605 | 0.334 | 0.117 |
| 14 | Malaysia | 3.278*** | 1.940** | 1.367 | 1.115 | 0.889 | 0.736 | 0.537 | 0.278 | 0.096 |
| 15 | Qantas | 2.252** | 1.687* | 1.456 | 1.638 | 1.773* | 1.296 | 0.852 | 0.667 | 0.156 |
| 16 | Shandong | 2.966*** | 1.724* | 1.912* | 1.387 | 1.036 | 0.748 | 0.576 | 0.526 | 0.684 |
| 17 | Singapore | 2.780*** | 1.662* | 1.549 | 1.213 | 0.835 | 0.923 | 0.729 | 0.66 | 0.801 |
| 18 | Southwest | 2.185** | 1.264 | 0.869 | 1.59 | 1.152 | 0.864 | 0.485 | 0.251 | 0.777 |
| 19 | Thai | 2.707*** | 3.319*** | 2.513*** | 1.760** | 1.262 | 0.902 | 0.719 | 0.269 | 0.763 |
| 20 | United | 1.307 | 2.501*** | 1.439 | 1.479 | 0.748 | 0.415 | 0.474 | 1.266 | 0.968 |

***,**,* indicates the rejection of the null hypothesis of non-causality at the 1%, 5% and 10%, respectively. The critical values at 1%, 5% and 10% are 2.33, 1.96 and 1.645, respectively.

Table A3: Causality in Variance for Full sample

|  | $\boldsymbol{H}_{\boldsymbol{0}}$**: EMV_ID does not Granger-cause:** | Quantiles | | | | | | | | |
| --- | --- | --- | --- | --- | --- | --- | --- | --- | --- | --- |
|  |  | 1 | 2 | 3 | 4 | 5 | 6 | 7 | 8 | 9 |
| 1 | American | 3.683*** | 2.163** | 1.486 | 1.11 | 0.978 | 0.72 | 0.436 | 0.226 | 0.079 |
| 2 | Ana | 3.558*** | 2.401*** | 2.465*** | 1.737* | 1.245 | 0.813 | 0.489 | 0.249 | 0.083 |
| 3 | Anaam | 0.208 | 0.256 | 0.358 | 0.46 | 0.32 | 0.303 | 0.342 | 0.361 | 0.369 |
| 4 | Asiana | 1.377 | 0.812 | 0.847 | 0.881 | 1.027 | 0.884 | 0.71 | 0.659 | 1.57 |
| 5 | Cathay | 2.763*** | 1.243 | 1.359 | 1.176 | 0.981 | 0.932 | 0.624 | 0.942 | 0.194 |
| 6 | China | 18.269*** | 7.089*** | 2.551*** | 0.587 | 0.238 | 0.279 | 0.166 | 0.206 | 0.297 |
| 7 | China east | 2.507*** | 1.638 | 1.221 | 0.951 | 0.872 | 0.813 | 0.549 | 0.282 | 0.097 |
| 8 | China south | 2.507*** | 1.638 | 1.221 | 0.951 | 0.872 | 0.813 | 0.549 | 0.282 | 0.097 |
| 9 | Delta | 3.447*** | 2.090** | 1.753* | 1.265 | 0.948 | 0.748 | 0.647 | 0.191 | 0.064 |
| 10 | Deutsche | 2.321*** | 1.153 | 1.127 | 0.637 | 0.89 | 1.224 | 0.803 | 0.647 | 0.143 |
| 11 | Eva | 2.783*** | 1.592 | 1.106 | 1.27 | 0.672 | 0.566 | 0.814 | 0.645 | 0.988 |
| 12 | France | 2.309** | 1.466 | 1.152 | 1.05 | 1.107 | 0.723 | 0.436 | 0.223 | 0.075 |
| 13 | Hainan | 8.691*** | 2.511*** | 0.525 | 0.265 | 0.328 | 0.552 | 0.818 | 0.368 | 0.163 |
| 14 | Malaysia | 4.110*** | 2.805*** | 2.268** | 1.619 | 1.203 | 0.944 | 0.632 | 0.53 | 0.626 |
| 15 | Qantas | 2.493*** | 1.458 | 2.506*** | 1.763* | 1.266 | 0.927 | 0.503 | 0.258 | 0.088 |
| 16 | Shandong | 6.254*** | 3.669*** | 2.718*** | 1.862* | 1.266 | 0.933 | 0.724 | 0.64 | 0.762 |
| 17 | Singapore | 4.784*** | 2.753** | 1.798* | 1.22 | 0.841 | 0.707 | 0.698 | 0.851 | 1.355 |
| 18 | Southwest | 0.693 | 1.281 | 1.968** | 1.784* | 1.985** | 2.150** | 2.029** | 1.836* | 1.454 |
| 19 | Thai | 3.387*** | 2.016** | 1.644 | 1.154 | 1.001 | 0.601 | 0.452 | 0.426 | 0.611 |
| 20 | United | 2.832*** | 1.604 | 1.073 | 1.494 | 1.158 | 1.041 | 0.877 | 0.978 | 0.757 |

***,**,* indicates the rejection of the null hypothesis of non-causality at the 1%, 5% and 10%, respectively. The critical values at 1%, 5% and 10% are 2.33, 1.96 and 1.645, respectively.

Table A4: Causality in Variance COVID-19 sample

|  | $\boldsymbol{H}_{\boldsymbol{0}}$**: EMV_ID does not Granger-cause:** | Quantiles | | | | | | | | |
| --- | --- | --- | --- | --- | --- | --- | --- | --- | --- | --- |
|  |  | 1 | 2 | 3 | 4 | 5 | 6 | 7 | 8 | 9 |
| 1 | American | 2.838*** | 2.116** | 1.469 | 1.119 | 1.201 | 0.893 | 0.48 | 0.249 | 0.087 |
| 2 | Ana | 2.409*** | 1.599 | 1.613 | 1.701 | 1.074 | 0.7 | 0.719 | 0.866 | 0.471 |
| 3 | Anaam | 2.402*** | 1.554 | 1.591 | 1.38 | 0.949 | 1.07 | 0.917 | 0.599 | 0.1 |
| 4 | Asiana | 3.091*** | 2.323** | 1.613 | 1.322 | 0.966 | 0.687 | 0.415 | 0.212 | 0.072 |
| 5 | Cathay | 3.399*** | 1.881* | 1.575 | 1.785* | 1.902* | 1.516 | 1.065 | 0.561 | 0.153 |
| 6 | China | 6.987*** | 3.413** | 1.792* | 1.44 | 0.838 | 2.757*** | 2.571*** | 0.761 | 0.395 |
| 7 | China east | 6.555*** | 3.846*** | 2.600*** | 1.858* | 1.378 | 1.071 | 0.909 | 0.586 | 0.644 |
| 8 | China south | 1.873* | 1.676* | 1.288 | 1.15 | 1.061 | 0.733 | 0.567 | 0.296 | 0.105 |
| 9 | Delta | 2.546*** | 2.673*** | 1.799* | 1.279 | 0.944 | 0.671 | 0.406 | 0.21 | 0.073 |
| 10 | Deutsche | 3.220*** | 2.551*** | 1.687* | 1.42 | 0.981 | 0.627 | 0.406 | 0.316 | 0.107 |
| 11 | Eva | 3.979*** | 2.340*** | 1.660* | 1.451 | 1.037 | 0.771 | 0.808 | 0.444 | 0.155 |
| 12 | France | 1.601 | 1.437 | 1.335 | 1.591 | 0.715 | 1.072 | 0.939 | 0.718 | 0.524 |
| 13 | Hainan | 2.728*** | 2.353*** | 1.667* | 0.918 | 0.702 | 0.705 | 0.803 | 0.615 | 0.117 |
| 14 | Malaysia | 3.110*** | 1.872* | 1.331 | 1.062 | 1.045 | 0.885 | 0.748 | 0.641 | 1.327 |
| 15 | Qantas | 2.078** | 1.2497 | 0.947 | 1.148 | 0.968 | 0.809 | 0.893 | 0.609 | 0.156 |
| 16 | Shandong | 4.003*** | 2.339*** | 1.835* | 1.730* | 1.246 | 0.922 | 0.792 | 0.264 | 0.092 |
| 17 | Singapore | 4.784*** | 2.753** | 1.798* | 1.22 | 0.841 | 0.707 | 0.698 | 0.851 | 1.355 |
| 18 | Southwest | 0.693 | 1.281 | 1.968** | 1.784* | 1.985** | 2.150** | 2.029** | 1.836* | 1.454 |
| 19 | Thai | 3.387*** | 2.016** | 1.644 | 1.154 | 1.001 | 0.601 | 0.452 | 0.426 | 0.611 |
| 20 | United | 0.561 | 1.085 | 1.27 | 2.384*** | 3.777*** | 5.076*** | 4.187*** | 2.911*** | 0.93 |

***,**,* indicates the rejection of the null hypothesis of non-causality at the 1%, 5% and 10%, respectively. The critical values at 1%, 5% and 10% are 2.33, 1.96 and 1.645, respectively.
