# Supplementary material for: Economic uncertainty of pandemic and international airlines behaviour
Source: PLoS One. 2022 May 26;17(5):e0266842. doi: 10.1371/journal.pone.0266842 (PMC9135306; doi:10.1371/journal.pone.0266842)
Supplement: Attachment — Submitted filename: Response to Reviewer Plos 1.docx [file pone.0266842.s002.docx]

Re-submission of manuscript entitled: **Economic uncertainty of pandemic and International airlines behavior**

**Manuscript ID:** PONE-D-21-38713

Dear Editor,

Thank you for reviewing our article with the above caption. We are very grateful for your constructive and valuable comments, and particularly for giving us the opportunity to make a revision. The paper has been revised to accommodate your comments.

EDITOR’s COMMENTS:

Comment 1: Please ensure that your manuscript meets PLOS ONE's style requirements, including those for file naming. The PLOS ONE style templates can be found at

<https://journals.plos.org/plosone/s/file?id=wjVg/PLOSOne_formatting_sample_main_body.pdf> and

<https://journals.plos.org/plosone/s/file?id=ba62/PLOSOne_formatting_sample_title_authors_affiliations.pdf>

**Response 1:**

Many thanks for the comment. We have revised the manuscript to accommodate this.

**Comment 2:** Please review your reference list to ensure that it is complete and correct. If you have cited papers that have been retracted, please include the rationale for doing so in the manuscript text, or remove these references and replace them with relevant current references. Any changes to the reference list should be mentioned in the rebuttal letter that accompanies your revised manuscript. If you need to cite a retracted article, indicate the article’s retracted status in the References list and also include a citation and full reference for the retraction notice.

***Response 2:*** *Thank you for your comment. The manuscript has been adjusted to ensure that the full reference of all in-text citations are well highlighted in the reference list. Also, due to the reviewer’s comments. Some new studies were cited such as,*

*Adekoya, O.B., & Oliyide, J.A. (2021). How COVID-19 drives connectedness among commodity and financial markets: Evidence from TVP_VAR and causality-in-quantiles techniques. Resources Policy, 70, 101898.*

*Fasanya, I.O., Oliyide, J.A., Adekoya, O.B., & Agbatogun, T. (2021c). How does economic policy uncertainty connect with the dynamic spillovers between precious metals and bitcoin markets? Resources Policy, 72.*

*Lintner, J. (1965). The valuation of risk assets and the selection of risky investments in stock portfolios and capital budgets. The Review of Economics and Statistics 47, 13-37.*

*Oliyide, J.A., Adekoya, O.B., & Khan, M.A. (2021). Economic policy uncertainty and the volatility and the volatility connectedness between oil shocks and metal market: An extension. International Economics, 167, 136-150.*

*Sharpe, W.E. (1964). Capital-asset prices- A theory of market equilibrium under conditions of risk. Journal of Finance, 19, 425-442.*

Comment 3: Thank you for stating the following financial disclosure:

"The authors received no specific funding for this work”

*Response 3: Thank you, Editor.*

Comment 4: Please clarify the sources of funding (financial or material support) for your study. List the grants or organizations that supported your study, including funding received from your institution.

*Response 4: “The authors received no specific funding for this work”*

Comment 5: State what role the funders took in the study. If the funders had no role in your study, please state: “The funders had no role in study design, data collection and analysis, decision to publish, or preparation of the manuscript.”

*Response 5: Thank you for your comment. (The authors received no specific funding for this work)*

Comment 6: If any authors received a salary from any of your funders, please state which authors and which funders.

Response 6:

The authors received no specific funding for this work

Comment 7: In your Data Availability statement, you have not specified where the minimal data set underlying the results described in your manuscript can be found. PLOS defines a study's minimal data set as the underlying data used to reach the conclusions drawn in the manuscript and any additional data required to replicate the reported study findings in their entirety. All PLOS journals require that the minimal data set be made fully available. For more information about our data policy, please see <http://journals.plos.org/plosone/s/data-availability>.

Response 7:

The airline stocks data employed in this study is owned by Refinitiv Eikon. The authors accessed the data with their institution’s membership. The authors had no special access privileges to the data others would not have. The data underlying the results presented in the study are available from Thompson Reuters DataStream Database (https:// eikon.thomsonreuters.com/index.html). While the data on infectious diseases uncertainty is freely available on <http://policyuncertainty.com/infectious_EMV.html>.

**REVIEWER’S COMMENTS:**

Comment 1: The article deals with a very interesting topic related to the prediction of the value of shares in times as complex as the current ones derived from the COVID-19 pandemic, and very especially, for the airline sector

*Response 1: Thank you for your kind words and your detailed review of our paper.*

Comment 2: The study is adequately justified, but in the specific case that is exposed of the airlines, it would be advisable to have a deeper justification in relation to the variables to be studied and even add a greater number of cases, whenever possible or for subsequent studies, therefore which in this case could be exposed as future lines of research. See, in this last sense, the following article:

Bousoño-Calzón, C., Bustarviejo-Muñoz, J, Aceituno-Aceituno, P., Escudero Garzas, J.J. (2019). On the Economic Significance of Stock Market Prediction and the No Free Lunch Theorem. IEEE ACCESS. 7, 75177-75188.

**Response 2:** *Your comment is highly appreciated. It is important mention that the selection of the airline stocks are based on data availability and on their two-letter designations from the International Air Transport Association (IATA). We have incorporated this in the data section. Also, we have suggest some critical research areas to future studies.*

Comment 3: The discussion between the results and the theoretical framework from which the objective is formulated has not really taken place. These aspects are not discussed, nor are the authors exposed in the theoretical framework cited in the discussion, and with this, the contribution of the article is not clearly established.

***Response 3:*** *Thank you so much for your constructive comment. We find this comment very valuable for the re-construction of our manuscript, hence we have revisited the whole manuscript. First of all, as seen in the fourth paragraph in the introduction section, we have highlighted the gap in the literature and subsequently derive the contribution thereof. Secondly, we briefly introduce the standard capital assets pricing model. In all, we revisited the discussion of the findings where the contribution of the study are adequately linked with capital assets pricing model.*
